# Comparison of diode laser – Oral tissue interaction to different wavelengths. In vitro study of porcine periodontal pockets and oral mucosa

**DOI:** 10.4317/medoral.23317

**Published:** 2020-01-22

**Authors:** Aida Gutiérrez-Corrales, Yudex Rizcala-Orlando, Paloma Montero-Miralles, Gerd Volland, Jose Luis Gutiérrez-Pérez, Daniel Torres-Lagares, Maria Angeles Serrera-Figallo

**Affiliations:** 1 DDS. Dental School. University of Seville, Spain; 2 DDS, PhD. Dental School. University of Seville, Spain.; 3 DMD, PhD. Dental School. University of Seville, Spain

## Abstract

**Background:**

The aim of this in vitro study was to evaluate the effect of diode lasers at different wavelengths and power settings in handmade incisions in periodontal pockets and in oral mucosa of porcine tissue considering thermal damage, necrosis and the affected area of the soft tissue.

**Material and Methods:**

Combining the following laser wavelengths, 445nm, 532nm (KTP), 810nm, 980nm, 1064nm and 1470nm, and a power range from 0.5W to 2.0W in a continuous wave mode (CW), we made handmade incisions in porcine periodontal pockets and oral mucosa. After histological processing, we measured the area of ​​lost tissue, the area of ​​thermal damage and the area of ​​necrosis. Then, we performed ANOVA to evaluate the difference between groups and two-way ANOVA to identify the influence of the laser-type variables and the power on the results.

**Results:**

We applied an ANOVA test to evaluate the results, where statistical analysis showed clear differences between the 1470nm and 810nm laser groups that refer to thermal damage and necrosis in the periodontal pocket surface. Regarding the oral mucosa surface, the 1064nm laser showed differences in the analysis of lost tissue. According to the applied power, all the variables we studied (lost tissue area, area of thermal damage and necrosis) showed higher values when using a power of 2.0W instead of 0.5W.

**Conclusions:**

According to our results, the 810nm diode laser for oral soft-tissue biopsy using power ranges between 0.5W and 2W would be the best choice to avoid thermal damage in peri-incisional margins.

** Key words:**Laser surgery, soft tissue, diode laser, KTP laser, thermal damage, necrosis.

## Introduction

Although the first articles describing the use of a laser technique in the oral cavity were introduced in the 1960s, laser therapy became a revolution in the field of dentistry in the 1990s. This is due to the fact that the FDA (Food and Drug Administration) of the US approved the first laser designed specifically for general dentists: Nd:YAG Laser 300, developed by Myers (1,2). Since then, there has been an increase in the number of available wavelengths from the Nd:YAG laser, including CO2, Nd:YAG, argon, various diode wavelengths (810nm, 940nm, 980nm and 1.064nm), erbium YAG, erbium chromium YSGG and potassium titanyl phosphate (KTP). These wavelengths are important because of how laser light is delivered to the surgical site and how it interacts with the tissue to reach different depths of penetration. For this reason, they have been promoted for many procedures, although they belong to different absorption values in the electromagnetic spectrum (3).

After more than 50 years of this initial experimental use in dentistry, as well as almost 30 years after its practical introduction in dental surgery, evidence has shown improved clinical outcomes in the use of lasers in dentistry procedures, which have acquired special importance for both the dentist and patient (4). Even with the many applications in periodontology, restorative and conservative dentistry, oral and maxillofacial surgery, aesthetic dentistry, orthodontics or even dental implants, soft-tissue treatment has become one of the most relevant applications due to its efficacy and safety. The advantages of laser surgery in comparison to a conventional technique for the clinician are, mainly, an excellent haemostasis with a relatively bloodless wound, greater precision, sterilization of the surgical area, minimal swelling and scarring and no suture needed. In conclusion, a controlled tissue resection reduces bleeding and difficulty of surgery on the lips, tongue, cheeks and sublingual regions. This technique also has some benefits for patients such as more confidence, serenity and better postoperative results with minimal pain (5-9).

The amount of energy absorbed during laser therapy depends on the wavelength and the biological tissue characteristics, such as pigmentation or water content. The main laser–tissue interaction is photothermic, which means that laser energy is transformed into heat, and by modifying different parameters, such as spot size, energy or time, we could obtain three different effects in the soft tissue: incision or excision, ablation or vaporization and haemostasis or coagulation (10-12).

On one hand, water, which is present in biological tissues like oral mucosa, absorbs many of the wavelengths of erbium and CO2 lasers, which means that light only penetrates a few microns into the target tissue. On the other hand, water allows deeper laser transmission of shorter wavelengths such as the diode or Nd:YAG lasers. It is important to consider that penetration of some wavelengths inside mucosa allows a tissue interaction that continues beyond the surgical field. This could lead to deep thermal necrosis of underlying tissue or even bone osteonecrosis (13). Since the KTP (potassium titanium phosphate) laser was introduced in soft-tissue surgery, it has become a very successful treatment option due to its great affinity for haemoglobin and oxihaemoglobin in comparison to Nd:YAG, wherein energy is absorbed in a superficial tissue level, thus avoiding deep-tissue penetration (14,15).

In addition to lasers in a solid or gas state, such as Er:YAG, Er:YSGG or CO2 lasers, diode lasers have been applied for dental treatments with many advantages, such as disinfectant ability or effectiveness in the coagulation of superficial injuries, providing a dry surgical field without risk of haemorrhage. As with some features, this energy penetrates better in pigmented substances (such as haemoglobin and melanin), and the 810nm, 940nm and 980nm wavelengths have been recognized, but 1064nm was rarely used. Recently, new visible wavelengths have appeared, such as systems that emit a laser blue radiation in the spectral range of 445nm or 532nm (16-18). Furthermore, although diode lasers are available with different wavelengths, according to some studies, the wavelength of 980nm is absorbed by water with a speed slightly higher than 810nm, which makes the 980nm diode laser potentially safer (19).

Many studies have been published comparing these wavelengths; unfortunately, without using the same parameters, comparisons are not possible. Due to differences in the physical properties of the tissues, the interaction between laser and tissue could not be properly evaluated. Otherwise, most recent studies have used high power settings to determine the thermal effect and necrosis in the cut tissue. The aim of this *in vitro* study was to discover how the low power settings of 0.5W and 2.0W in a continuous wave (CW) mode can affect the tissue, as well as which effect could be obtained in porcine gingiva depending on the wavelength used. For this reason, through analysis of the currently available wavelengths, we evaluated their ability to perform cuts in mucosa and periodontal pockets in porcine gingiva.

The purpose of this study was to determine which technique (combining the type of laser, wavelength and power) shows the best results in the efficacy and safety for the treatment of oral soft tissue.

## Material and Methods

- Laser wavelengths and systems

We used different laser wavelengths of 445nm (FOX IV), 532nm (NuvoLas), 810nm (FOX), 980nm (FOX), 1064nm (FOX) and 1470nm (WOLF) (all systems provided by A.R.C. Laser GmbH, Nuremberg, Germany) at a power range of 0.5W to 2.0W in increments of 0.5W. Laser radiation was emitted in a CW mode.

We carried out laser transmission through a 300 micrometre bare fibre (with a 280 micrometre core diameter). We fixed the fibre in a handpiece typically used for surgical applications in dentistry. Also, we fixed the inclination angle at 30° using a curved tip attached to the handpiece. Fibre projection was adjusted at 3mm.

- Specimens

We performed laser incisions in porcine periodontal pockets and oral mucosa. Therefore, we dissected freshly slaughtered pig jaws, specifically the region of the first and second molar. This sample consisted of bone, teeth and attached gingiva. Then, we performed 24 samples with a size of 40x30mm. We marked the buccal area of the samples with suture material to ensure a clear allocation after histological preparation.

- Laser incisions

We properly cleaned the fibre before each laser incision. Also, we measured output power at the fibre tip with a powermeter to ensure all the incisions were set with equal parameters. For all incisions, we used a non-initialized bare fibre, which resulted in a pure tissue interaction, due to the laser radiation itself, without the hot effect of initialized fibres. We applied the laser twice in a paramarginal incision (10mm and 12mm apical to the free gingival margin of first and second molars) in a CW mode with a 300μm optical fibre. Regarding the periodontal pocket, we made cuts in this area of ​​the first and second molars. Thus, in each specimen, we obtained four cutting images in the oral mucosa and four in the periodontal pocket. We obtained four images (n = 4) for each combination of laser and power. Each type of laser had 16 images.

- Histology

**Figure 1:**
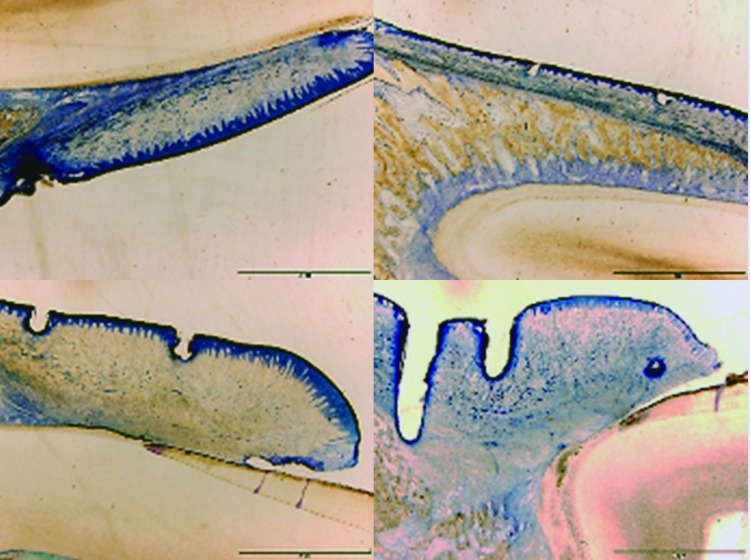
Different images of histological sections. Top left: 1470nm / 0.5W; Top right: 980nm / 1W; Bottom left: 810nm / 1.5W; Bottom right: 532nm / 2W.

We preserved specimens in a 100ml solution with 70% ethanol. Then, we made histological sections through the EXAKT system (without decalcification). We stained sections with toluidine blue and prepared them for analysis under an optical microscope (Fig. 1).

We evaluated the effect according to the following: 1) thermal damage in oral mucosa and periodontal pocket (area and perimeter); 2) necrosis in oral mucosa and periodontal pocket (area and perimeter); and 3) affected area in oral mucosa and periodontal pocket surfaces.

- Statistics

We summarized the data in measures of centrality and dispersion (mean and standard deviation) for each laser and for each combination of laser and power, and we performed a one-way ANOVA to identify statistically significant differences between the groups. Also, we applied a two-way ANOVA to identify which variable or variables (e.g., power, type of laser) influenced the outcome variables measured in this study.

## Results

We obtained the results of this study through the application of different laser wavelengths on soft tissues at different power ranges. We analysed the effects on the cervical and apical areas of ​​treated pockets jointly by the type of laser (Table 1), the type of power (Table 2) or separately (Table 3).

**Table 1:**
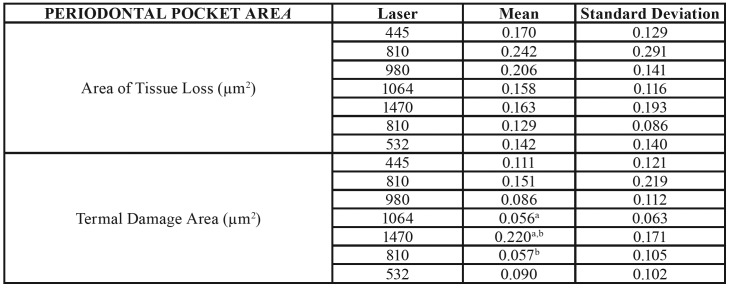
(Superior) Area of Tissue Loss, Termal Damage Area, Necrosis Area, Total Damage Area and Ratio Total Damage Area / Area of Tissue Loss by laser used in the periodontal pocket area. (Inferior) Area of Tissue Loss, Termal Damage Area, Necrosis Area, Total Damage Area and Ratio Total Damage Area / Area of Tissue Loss by laser used in the oral mucosa area.

**Table 1 cont.**
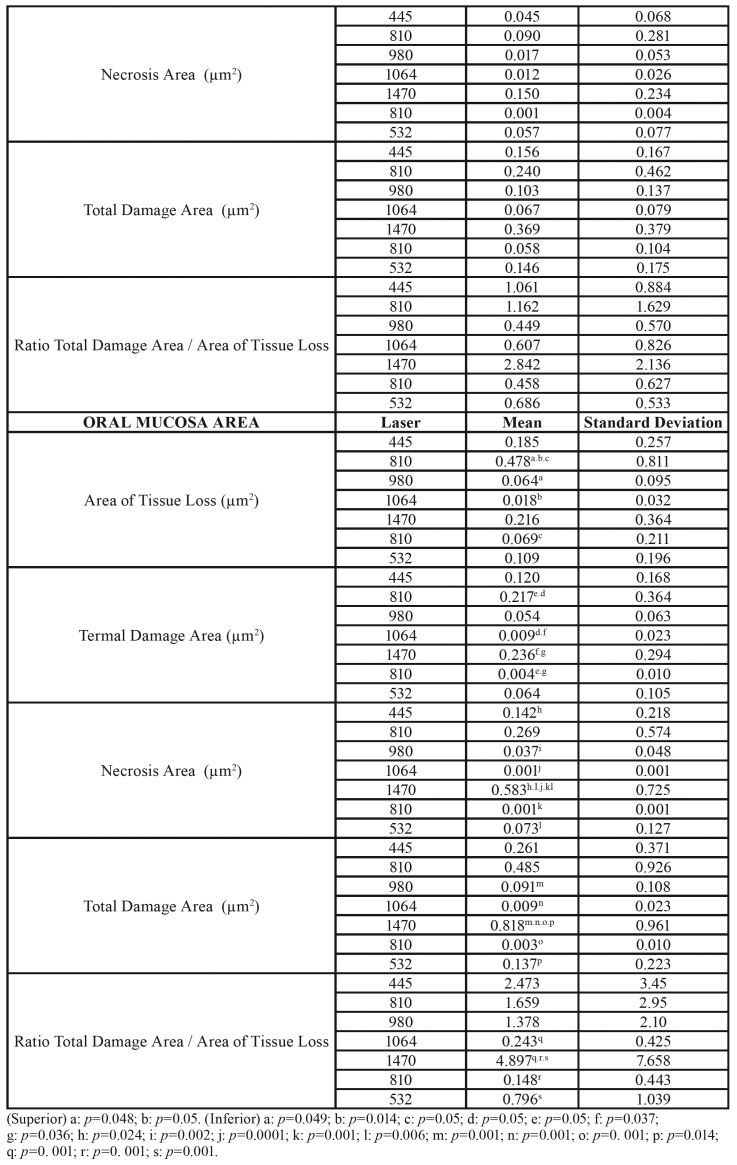
(Superior) Area of Tissue Loss, Termal Damage Area, Necrosis Area, Total Damage Area and Ratio Total Damage Area / Area of Tissue Loss by laser used in the periodontal pocket area. (Inferior) Area of Tissue Loss, Termal Damage Area, Necrosis Area, Total Damage Area and Ratio Total Damage Area / Area of Tissue Loss by laser used in the oral mucosa area.

**Table 2:**
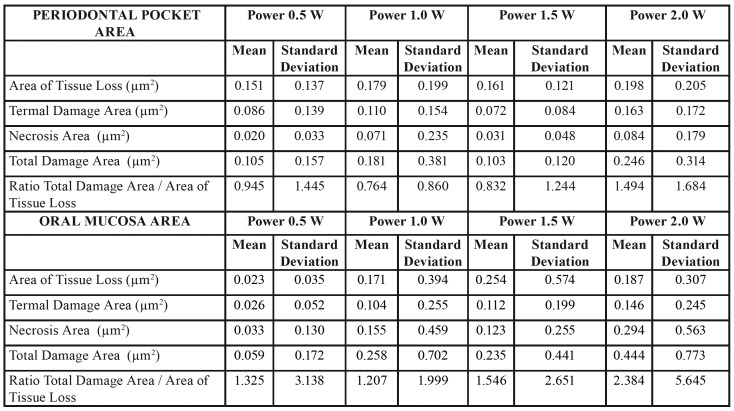
(Superior) Area of Tissue Loss, Termal Damage Area, Necrosis Area, Total Damage Area and Ratio Total Damage Area / Area of Tissue Loss by power used in the periodontal pocket area. (Inferior) Area of Tissue Loss, Termal Damage Area, Necrosis Area, Total Damage Area and Ratio Total Damage Area / Area of Tissue Loss by power used in the oral mucosa area.

**Table 3:**
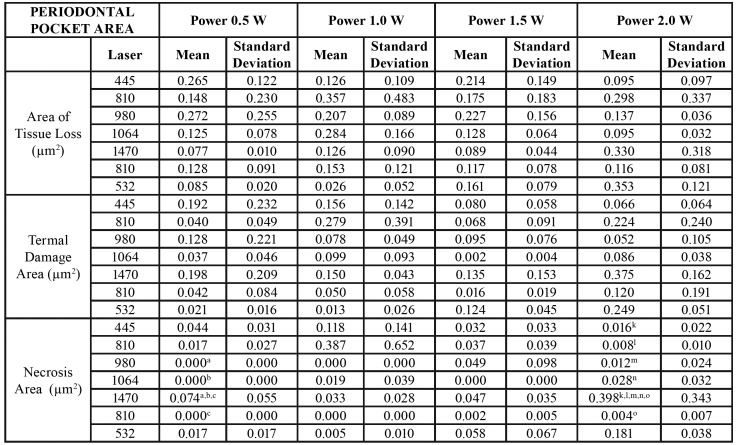
(Superior) Area of Tissue Loss, Termal Damage Area, Necrosis Area, Total Damage Area and Ratio Total Damage Area / Area of Tissue Loss by laser and power used in periodontal pocket area. (Inferior) Area of Tissue Loss, Termal Damage Area, Necrosis Area, Total Damage Area and Ratio Total Damage Area / Area of Tissue Loss by laser and power used in the oral mucosa area.

**Table 3 cont.**
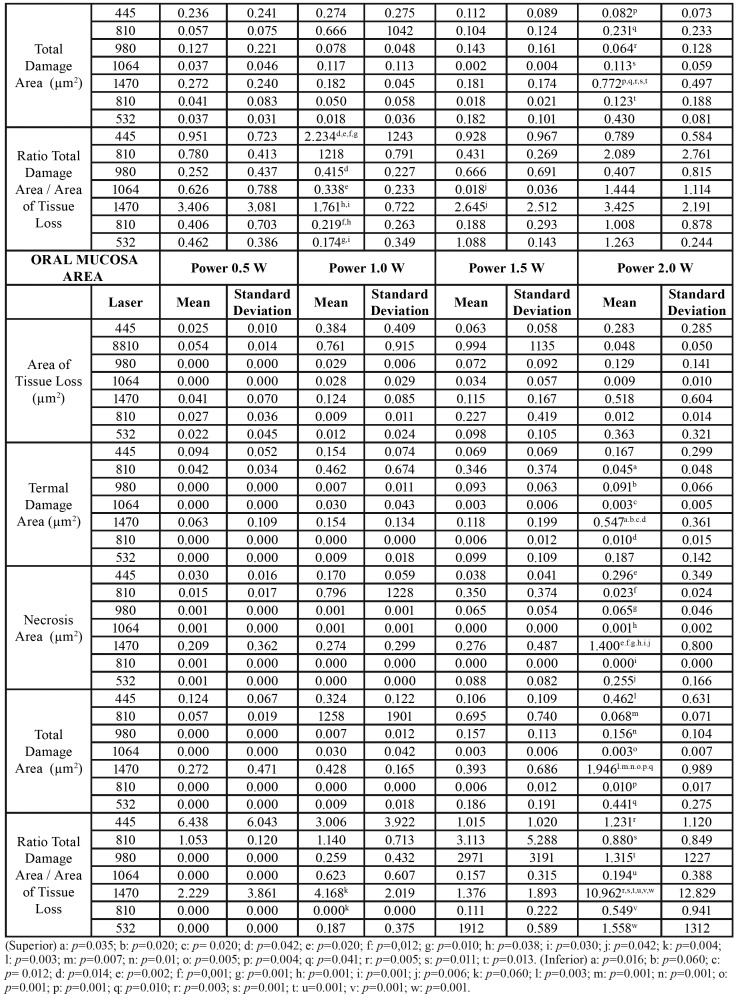
(Superior) Area of Tissue Loss, Termal Damage Area, Necrosis Area, Total Damage Area and Ratio Total Damage Area / Area of Tissue Loss by laser and power used in periodontal pocket area. (Inferior) Area of Tissue Loss, Termal Damage Area, Necrosis Area, Total Damage Area and Ratio Total Damage Area / Area of Tissue Loss by laser and power used in the oral mucosa area.

- Periodontal pocket effects

In relation to the lost tissue area once the procedure was completed, we found no significant differences for any of the studied laser groups. The 1470nm laser group (0.216µm2 ± 0.364) showed a greater tissue loss, and we observed the least amount of lost tissue in the 1064nm laser group (0.064µm2 ± 0.095). In addition, the area of thermal damage was greater in the 1470nm laser group (0.236µm2 ± 0.294), which showed significant differences with lasers that produced less thermal damage (1064nm - (0.009µm2 ± 0.023) and 810nm - (0.004µm2 ± 0.010)).

We found significant differences between the 1470nm laser group and the other studied groups. The area of necrosis was higher in the 1470nm laser group (0.583 µm2 0.725 ±) and less in the 810nm laser group (0.001 µm2 ± 0.001). The area of total damage (sum of area of thermal damage and necrosis) showed similar results between different types of studied lasers (except between the 1470nm and 445nm laser groups). This variable was higher in the 1470nm laser group (0.818µm2 ± 0.961) and minimal in the 810nm laser group (0.003µm2 ± 0.010).

To evaluate the way through which both variables (type of laser and power) could influence the results, we applied an ANOVA test on the lost tissue area, area of thermal damage, necrosis and lost tissue (the rest of the variables were ignored, including area of total damage and ratio between this last variable and lost tissue area, since they were variables calculated from the previous data). Analysis of the ANOVA test showed the following results: The lost tissue area was related to the type of laser and the applied power (*p* = 0.036) (R2 = 0,414); and the area of thermal damage was related to the laser used and applied power (*p* = 0.030) (R2 = 0.448). Finally, the area of necrosis was equal, but even the greater power was related to the type of laser used and the power used (*p* < 0.0001) (R2 = 0.564).

- Oral mucosa effects

Regarding the results of the laser type, in relation to the lost tissue area, we did not find significant differences in any studied laser group (n = 16 in each laser type) (n = 4 per each power used). The 1470nm laser showed a greater tissue loss (0.216µm2 ± 0.364), and the 1064nm laser showed the least amount of lost tissue (0.064µm2 ± 0.095).

The area of thermal damage was greater in the 1470nm laser group (0.236µm2 ± 0.294), showing a significant difference with lasers that caused less thermal damage (1064nm - (0.009µm2 ± 0.023) and 810nm - (0.004µm2 ± 0.010)). The area of necrosis was higher in the 1470nm laser group (0.583 µm2 0.725 ±) and minimal in the 810nm laser group (0.001µm2 ± 0.001). In summary, we found significant differences mainly between the 1470nm laser and the other studied groups. In the area of total damage (sum of the area of thermal damage and necrosis), we obtained similar differences between different types of studied lasers (except between the 1470nm and 445nm laser groups). This variable was higher in the 1470nm laser (0.818µm2 ± 0.961) and minimal in the 810nm laser (0.003µm2 ± 0.010).

Finally, when we calculated the ratio of the lost tissue area and total damaged area, the ratio was higher for the 1470nm laser group (4,897µm2 ± 7.658) and minimal for the 810nm laser group (0.148µm2 ± 0.443). According to the studied variables and applied power (0.5W, 1.0W, 1.5W and 2.0W, n = 28 by each power), we did not find significant differences, although patterns have a clearly upward trend, even if they were not clearly identified as linear. Nevertheless, experimentation and biological variability could explain this fact. The variables we studied included the following: lost tissue area, area of thermal damage, necrosis, total damage and ratio of total damaged area through higher values when using a power of 2.0W rather than 0.5W.

The lost tissue area was not related, in the two-way ANOVA analysis, to the type of laser or to the power. The area of ​​thermal damage was related to the type of laser used (*p* < 0.027, R2 = 0.346) but not to the power used. Finally, the area of ​​necrosis was also related to the type of laser used (*p* < 0.025, R2 = 0.435) but not to the power used.

## Discussion

Since the introduction of the ruby laser, other types of lasers have been used over the years, such as argon, carbon dioxide, neodymium:yttrium-aluminium-garnet (Nd:YAG), diode and erbium (Er:YAG and Er,Cr:YSGG) lasers with different and specific applications, but all of them provide multiple advantages (4). However, the main disadvantage of the laser is that it could create thermal damage to the target tissue through a photothermal effect. Das *et al*. (20) studied the laser-induced soft-tissue damage using 3D digital microscopy and suggested that the energy that increases temperature at the point of incidence (>100°C) causes vaporization, which could explain the laser effect in soft tissue. For this reason, it could be said that lasers cut by heating the tissue.

A side effect of this thermal reaction is an increase in the temperature of surrounding tissues, and this can create permanent or reversible damages. In our study, we considered evaluating thermal damage (area and perimeter), necrosis and the affected area. Our results showed that the extent of this damage is due to both wavelength and laser settings. To carry out this study, we used KTP and diode lasers. Different fields of dentistry such as oral surgery, endodontics, dental bleaching and periodontology have used the KTP laser (532nm, also called the doubled-frequency Nd:YAG laser). This laser has a visible green emission that is strongly absorbed by oxyhaemoglobin, which means a higher thermal effect in vascularized tissues, so lower levels of energy and fluence can be used to cut vascularized tissues, or even the output can be continuous or pulsed, depending on the application (21).

Romeo *et al*. (21) evaluated the histological peripheral damage caused by the application of the KTP laser during oral soft-tissue biopsy procedures in pig cadaver tongues, where the KTP laser demonstrated surgical effectiveness and caused little peripheral damage to the cut edges. In our study, we obtained the same results with the KTP laser (532nm), where no thermal damage or necrosis was presented in handmade incisions. Even in cervical and apical surfaces, the KTP laser application was made without affecting the surrounding area, which means that this type of laser is a safety choice to carry out surgical procedures.

In another study on the histological evaluation of *in vivo* margin biopsies, Romeo *et al*, analysed diode and KTP lasers and concluded that both tested lasers permitted sure histologic diagnosis, which means the histological artefacts can be controlled, especially when lower settings were applied (22). Referring to this, when we carried out an ANOVA test, we confirmed that the lost tissue area was related to the type of laser and the applied power (*p* = 0.036) (R2= 0,414), as well as the area of thermal damage (*p* = 0.030) (R2 = 0.448), but in contrast, necrosis results were equal.

In contrast, in a retrospective study, Angiero *et al*. (23) evaluated the thermal damage of 608 specimens of soft tissue with a diode laser from the oral cavity, and the results showed that a diode laser could induce serious thermal effects in small lesions (mean size below 3mm). Nevertheless, the authors suggested that specimens taken *in vivo* must have a minimum diameter of 5mm in order to have a reliable reading of histological samples. In this case, it is advisable to remember that tissues *in vivo*, compared to those ex vivo, are characterized by a higher concentration of liquid and that normal or pathological amounts of blood could explain the damage that tissue obtained using a diode laser, and this is in contrast to our study. Our results also showed thermal damage using a diode laser but mainly when we applied the 1470nm wavelength (0.236µm2 ± 0.294) in comparison with (1064nm - (0.009µm2 ± 0.023) and 810nm - (0.004µm2 ± 0.010)) laser groups with less thermal damage.

Whereas Erbium lasers work thermomechanically, all diode lasers have thermal effects on tissues, as well as in lower power ranges such as 0.5W to 2.0W, which could have a reproducible effect on treated gingiva. In relation to the power ranges applied in our study (0.5W, 1.0W, 1.5W and 2.0W), we did not find significant differences, although patterns were clearly ascending: we studied variables through higher values when using a power of 2.0W rather than 0.5W. This is due to the fact that energy is greatly absorbed by soft tissue and poorly absorbed by teeth and bones. This specific wavelength (810-980nm) is not only absorbed by water (although less than the CO2 laser wavelength) but also by other chromophores such as melanin and, in particular, oxyhemoglobin.

A diode laser is a semiconductor that uses solid-state elements, such as gallium, arsenide, aluminum and indium to change electrical energy into light energy. The main advantage of this laser is safety (avoiding tissue damage) in surgery procedures, even when it is used by contact or in a very close distance due to its beam escape (24). Furthermore, diode lasers have the ability to cut tissue to ensure coagulation and haemostasis and have a higher tissue ablation capacity in comparison to other laser systems. Many authors such as Akbulut *et al*. (25) and Merigo *et al*. (26) have reported a high percentage of successful soft-tissue lesions of the oral cavity using a diode laser. Although two wavelengths (810nm and 980nm) have been used the most in dental procedures utilizing diode lasers, other wavelengths have been proposed in the literature. In accordance with the results of Bostanciklioglu *et al*. (27), who evaluated the effect of laser irradiations at different wavelengths (660nm, 810nm, 980nm and 1064nm), Braun *et al*. (28) analysed the efficiency of a soft-tissue incision, so we could suggest that a 810nm diode laser wavelength is the best wavelength for soft-tissue surgical procedures based on our results. In addition to this, Fornaini *et al*. (19) and Usumez *et al*. (29), who compared the diode laser’s effects on soft-tissue surgery, revealed that the best-quality cut and the lowest temperature increased in specimens obtained with the shortest laser wavelength.

As clinicians, we must remember the advantages that laser therapy entails for our patients and that, according to the literature, the diode laser has proven to be the best option for soft-tissue surgical treatments. Thermal damage or even necrosis could appear if some parameters are not perfectly adjusted depending on the type of lesion and surgical procedure, which could delay healing and create greater discomfort among patients. According to our results and several studies, the best choice to avoid thermal-damaged tissues in peri-incisional margins is to use an 810nm diode laser for an oral soft-tissue biopsy using power ranges between 0.5W and 2W.
